# Respondent-driven sampling on the Thailand-Cambodia border. II. Knowledge, perception, practice and treatment-seeking behaviour of migrants in malaria endemic zones

**DOI:** 10.1186/1475-2875-10-117

**Published:** 2011-05-09

**Authors:** Piyaporn Wangroongsarb, Wichai Satimai, Amnat Khamsiriwatchara, Julie Thwing, James M Eliades, Jaranit Kaewkungwal, Charles Delacollette

**Affiliations:** 1 Bureau for Vector-borne Diseases, Ministry of Public Health, Bangkok, Thailand; 2 Center of Excellence for Biomedical and Public Health Informatics (BIOPHICS), Bangkok, Thailand; 3 Center for Disease Control and Prevention, Atlanta, USA; 4 World Health Organization, Mekong Malaria Programme, c/o Faculty of Tropical Medicine, Mahidol University; 420/6, Rajvithi Rd, Bangkok 10400, Thailand

## Abstract

**Background:**

Population movements along the Thailand-Cambodia border, particularly among highly mobile and hard-to-access migrant groups from Cambodia and Myanmar, are assumed to play a key role in the spread of artemisinin resistance. Data on treatment-seeking behaviours, knowledge and perceptions about malaria, and use of preventive measures is lacking as characteristics of this population prevent them from being represented in routine surveillance and the lack of a sampling frame makes reliable surveys challenging.

**Methods:**

A survey of migrant populations from Cambodia and Myanmar was implemented in five selected rural locations in Thailand along the Thai-Cambodian border using respondent driven sampling (RDS) to determine demographic characteristics of the population, migratory patterns, knowledge about malaria, and health-care -seeking behaviours.

**Results:**

The majority of migrants from Myanmar are long-term residents (98%) with no plans to move back to Myanmar, understand spoken Thai (77%) and can therefore benefit from health messages in Thai, have Thai health insurance (99%) and accessed public health services in Thailand (63%) for their last illness. In comparison, the majority of Cambodian migrants are short-term (72%). Of the short-term Cambodian migrants, 92% work in agriculture, 18% speak Thai, 3.4% have Thai health insurance, and the majority returned to Cambodia for treatment (45%), self-treated (11%), or did not seek treatment for their last illness (27%).

**Conclusion:**

Most highly mobile migrants along the Thai-Cambodia border are not accessing health messages or health treatment in Thailand, increasing their risk of malaria and facilitating the spread of potentially resistant *Plasmodium falciparum *as they return to Cambodia to seek treatment. Reaching out to highly mobile migrants with health messaging they can understand and malaria diagnosis and treatment services they can access is imperative in the effort to contain the spread of artemisinin-resistant *P. falciparum*.

## Background and rationale

The Greater Mekong Sub-region (GMS) is known as the global epicenter of *Plasmodium falciparum *resistance to anti-malarial drugs. Studies in the GMS over the last five years show an increased proportion of patients with delayed parasite clearance time when artemisinin monotherapies and combinations are used to manage *P. falciparum *infections. Therapeutic efficacy studies have identified areas along the Cambodia-Thailand border with frequently documented artemisinin resistance [1,2]. In response, a strategy to contain artemisinin-resistant parasites in south-east Asia was developed, and is now being implemented by the Ministries of Health in Thailand and Cambodia with technical leadership support from the World Health Organization and funding from the Bill and Melinda Gates Foundation [3,4]. The goal of the containment strategy is to reduce selection pressure and ultimately eliminate resistant *P. falciparum *strains in the 17 provinces of the Cambodia-Thailand border. One of the seven objectives of the containment project is to increase access to and use of malaria services and commodities by migrant and mobile populations.

Migrant and mobile populations along the border have historically facilitated the spread of resistant parasites to other countries and regions [5-7]. Substantial population movement across the Thai-Cambodian border due to political upheavals of the 1970s through the 1990s, the movement of military forces, and gem-mining and forestry activities have all brought partially immune or non-immune populations into close proximity to high transmission forested areas. In addition, there has been movement of migrant labour from Myanmar to the provinces on the Thai-Cambodian border, often from areas of high transmission. In a retrospective study in Thailand [8], it was found that the Thailand-Myanmar and Thailand-Cambodia border areas, locations with high numbers of migrant workers, had the highest incidence rates for malaria including *P. falciparum*, *Plasmodium vivax*, and mixed species infections.

The Bureau of Vector Borne Diseases (BVBD) in Thailand classifies migrants as M1 or M2; M1 are migrants who have been in Thailand for more than six months, and M2 are migrants who have been in Thailand less than six months [5]. Most M1s are registered with the Ministry of Labour (MOL), which gives them the right to remain in Thailand for a prescribed period of time (typically 1-2 years) and enables them to freely access the formal Thai healthcare system. M2s are often highly mobile, and are less likely to have registered with the MOL, though in border provinces such as Chantaburi, Trat, and Sa Kaeo, the provincial government gives permits at border crossings to enter that district of Thailand for one to seven days, which can be extended by returning to the border crossing for re-authorization. Both short- and longer-term registration procedures, however, are not consistent over time and space and depend on workforce needs and political orientations of governments in place. In principle, M2s do not have any claim to utilize the formal Thai healthcare system (other than the services provided by malaria clinics or employers willing to do so) and undocumented migrants can be arrested and deported at any time. Nonetheless, some are able to receive treatment at government health care facilities at the border, but this is inconsistent [9].

According to routine malaria surveillance in Thailand [10], non-Thais bear a disproportionately high proportion of the malaria burden, especially among M2 migrants. This situation presents serious problems for malaria control in Thailand; it compromises the achievements obtained by the Thai Health system through repeated re-introduction of malaria parasites into Thailand from migrant populations and it threatens significant numbers of migrant workers and Thai citizens with illness and death [5]. Moreover migrant workers are reluctant to miss work when ill, and many are not protected by laws or public social measures. This poor access to care and resulting delay in treatment -seeking puts them at risk for more severe illness and may contribute to drug resistance and higher disease transmission levels on international borders [11].

The containment project has undertaken specific activities to ensure access to preventive and curative malaria services for migrants including those who are undocumented in Thailand. These efforts are limited by the lack of data on migrant workers. The goals of this study were, therefore, to determine the proportions of settled and mobile migrant workers (including those undocumented) along the border, and to explore the knowledge, perceptions and practices as well as the treatment-seeking behaviours of the migrant and mobile populations so as to develop action plans to better target malaria prevention and treatment interventions.

## Methods

### Study area and population

**Figure 1:**
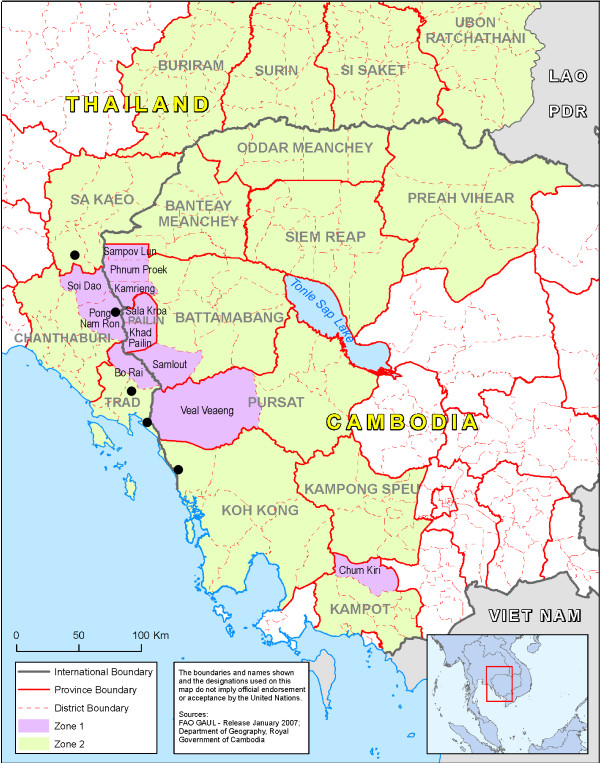
**Location of the 5 study sites**. Falciparum resistance to artemisinin has been documented in the hotspot Zone 1 where intensive containment operations are ongoing.

Three out of the seven Thai provinces targeted by the containment project were chosen along the Thailand-Cambodia border based on their large migrant populations to target both migrants from Cambodia and from Myanmar [12]. Five study sites were chosen: two in communities that had a high proportion of migrants from Myanmar and three with a high proportion of Cambodian migrants close by areas where artemisinin resistance to falciparum malaria has been first documented [2]. The study population included both M1 and M2 migrants. The survey was conducted from September to December 2009 in locations in Thailand on the border with Cambodia (Figure 1).

### Respondent driven sampling and recruitment

Standard cross-sectional and household survey methods were inadequate to obtain representative information due to the lack of a sampling frame, thus a respondent-driven sampling (RDS) methodology was used. Respondent-driven sampling was developed for hidden and hard to reach populations; the method relies on members of the target community to recruit other members of the community to participate [13,14]. While populations typically studied using respondent-driven sampling are urban, and have included injection drug users and sex workers, migrants may be similarly hidden, mobile, and difficult to sample [15].

Health care workers and survey staff from each study area were trained in RDS survey methodology. The teams then chose six initial participants, or "seeds" in the target community at each study site. These seeds received three uniquely numbered and identifiable coupons to recruit other participants in the community. Those participants, once interviewed, received 2-3 coupons to recruit additional participants, and the survey continued in this way until the required sample size was reached. Each generation of recruits is referred to as a "wave". Information regarding the size and depth of each person's social network was collected, as well as information regarding persons recruited and any refusals. Small incentives were given for both participation (being interviewed), and recruitment (recruiters were given an incentive for each of their 2-3 recruits that completed the interview process).

Sample sizes were calculated separately for migrants from Myanmar and migrants from Cambodia to account for the non-overlapping social networks of these two groups, requiring an approximate sample size of 900 participants for each of the two groups for a total of 1800 participants. A fuller description of the sampling methodology is described elsewhere [16].

### Ethical considerations

The protocol was reviewed by Members of the Communicable Diseases Department of the Ministry of Health and to be exempt from full Institutional Review Board review. Due to the sensitive nature of identity in populations for which RDS methodology is used, consent signed by the participant is not obtained. Following careful explanation of the survey, eligible participants were given the consent form to read or, if necessary, the consent form was read to the survey participant by project staff. All questions were addressed and consenting participants verbally stated that they understood and agreed to all of the items contained in the consent. Following this, a project staff member signed the consent form in the appropriate space.

### Data management and oversight

The data collected in the questionnaire included socio-demographics, migratory patterns, work history, health-seeking behaviour, knowledge about malaria, malaria prevention activities, and access to health messages. The coupon management system was developed by BIOPHICS (Mahidol University, Bangkok) and implemented for use at each survey site. Survey forms were designed and faxed to BIOPHICS via Datafax data management system. Data quality was checked and reconciled with the coupon management system. Supervisory visits by BVBD staff with technical support from WHO and BIOPHICS were conducted for the first two weeks of the survey, and at intermittent intervals during the course of implementation.

### Statistical analysis

Analyses were performed using the Respondent Driven Sampling Analysis Tool v. 5.6.0(RDSAT) [17]. RDSAT weights each variable by network size of each individual. Analysis was stratified by categorization of each migrant as M1 or M2, determined by whether the respondent had been in Thailand six or more months, or less than six months, respectively. The social network size was defined as all migrants living in the same community that the participant knew by first name or vice versa, and with whom they had met in the previous month. A fuller description of the statistical analysis is described elsewhere [16].

## Results

### Recruitment

**Figure 2:**
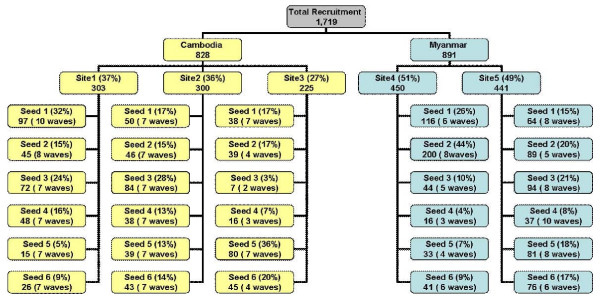
**Recruitment methodology**.

Among Cambodian migrants, study staff were able to identify 12 M1 (residing in Thailand at least six months) and six M2 (residing in Thailand less than 6 months) to serve as the 18 seeds. Despite intensive search, only one migrant from Myanmar who had resided in Thailand less than six months was identified to serve as a seed, thus there were 11 M1 and one M2 who served as the 12 seeds in the population from Myanmar. The 18 seeds from Cambodia recruited a total of 828 Cambodian migrants (350 M1, 475 M2, 3 not determined), and the 12 from Myanmar recruited migrants, recruited a total of 891 migrants (871 M1, 19M2). The recruitment by seed and site is demonstrated in Figure 2. The greatest number of recruits from any one seed was 200, while the greatest number of waves was 10. The homophily analysis is reported elsewhere [16], and demonstrated that the networks of short and long term migrants were integrated, both from Cambodia and Myanmar.

### Demographics

Among Cambodian migrants, 28% were M1 and 72% were M2. A greater proportion of M2 than M1 migrants were under 25 years of age (50% vs. 39%), male (65% vs. 52%), single (38% vs. 31%), and without any formal education (35% vs. 22%), though only lack of formal education reached statistical significance. A smaller proportion of M2 compared to M1 could speak Thai (18% vs. 56%) or read Thai (2% vs. 14%); both of these were statistically significant. Khmer was the first language of all but a few (Table 1).

**Table 1:** Weighted analysis of demographic characteristics of migrants

Variables	Cambodian		Myanmar	
	
	M1* n = 350 % (95% CI)	M2 ** n = 475 % (95% CI)	M1 * n = 871 % (95% CI)	M2 ** n = 19 % (95% CI)
**Total**	28.3% (25.5-33.3)	71.7% (66.7-74.5)	97.3% (95.8-98.6)	2.7% (1.4-4.2)
**Age**				
≤ 25 years	38.8% (31.6-46.5)	50.1% (45.7-54.6)	48.8% (45.4-53.1)	60.1% (39.4-90.1)
26 - 35 years	31.6% (25.4-38.1)	27.1% (23.2-31.1)	29.8% (26.0-32.8)	16.0% (0.0-35.4)
36 - 45 years	18.8% (14.2-24.5)	19.0% (15.4-22.9)	15.2% (13.2-17.9)	2.9% (0.0-5.3)
>45 years	10.8% (7.0-14.4)	3.8% (2.0-6.2)	6.2% (4.4-7.7)	21.0% (0.0-39.5)
**Gender**				
Male	51.5% (45.7-60.9)	64.6% (60.9-69.5)	56.5% (52.7-60.3)	38.1% (9.9-67.0)
Female	48.5% (39.1-54.3)	35.4% (30.5-39.1)	43.5% (39.7-47.3)	61.9% (33.1-90.2)
**Marital Status**				
Single	30.9% (22.5-37.4)	38.0% (33.1-42.2)	32.5% (28.6-36.3)	53.0% (27.7-83.4)
Married	61.7% (55.9-70.4)	58.1% (53.3-63.1)	65.8% (62.1-69.8)	45.7% (16.6-72.4)
Married, not living together	2.0% (0.4-4.0)	1.2% (0.3-2.7)	0.6% (0.3-0.9)	---
Divorced	1.0% (0.0-1.9)	1.2% (0.2-2.0)	0.4% (0.0-0.8)	---
Widowed	4.4% (2.1-6.7)	1.5% (0.5-2.8)	0.7% (0.3-1.2)	---
**Education level**				
No education	22.3% (17.7-27.5)	34.7% (30.3-39.5)	33.1% (29.4-36.8)	39.8% (12.3-62.5)
≤Primary School	58.1% (52.4-65.3)	52.2% (48.1-56.5)	62.5% (58.9-66.3)	60.2% (37.6-87.7)
>Primary School	19.6% (14.3-23.6)	20.5% (16.8-24.9)	4.3% (2.9-5.9)	---
**Ethnic Group**				
Karen	---	---	1.3% (0.3-2.5)	---
Mon	---	---	86.7% (83.6-89.7)	100%
Burmese	---	---	11.8% (.1-14.8)	---
Other	---	---	0.2 (0.0-0.5)	---
**Languages spoken**				
Thai	55.7% (48.9-63.0)	17.5% (14.1-20.4)	76.9% (74.2-80.0)	15.8% (4.0-36.3)
Khmer	99.6% (99.2-100)	99.7% (99.3-100)	0.3% (0.0-0.5)	---
Burmese	---	---	54.5% (51.0-58.5)	69.2% (42.1-93.1)
Mon	---	---	93.8% (81.6-95.9)	76.9% (49.2-100)
Karen	---	---	1.6% (0.6-3.0)	---
**Languages read**				
Thai	13.5% (9.4-17.9)	2.3% (0.9-3.9)	4.7% (3.1-6.2)	---
Khmer	75.9% (70.6-80.9)	64.0% (59.5-69.1)	---	---
Burmese	---	---	45.1% (41.3-49.4)	47.9% (24.6-81.0)
Mon	---	---	31.9% (28.2-35.6)	21.0% (7.6-43.5)
Karen	---	---	0.3% (0.0-0.8)	---

* Has lived in Thailand for 6 or more months; ** Has lived in Thailand for less than 6 months

Among the migrants from Myanmar, 98% were M1 and 2% were M2, so almost all were long standing residents. While the number of M2 was so small as to render meaningful comparisons impossible, among M1, 49% were under 25 years of age, 57% were male, 33% were single, and 33% had no formal education. Most (77%) could speak Thai, but only 5% could read Thai. While 55% spoke Burmese, only 12% identified themselves as Burmese. The majority (87%) identified themselves as Mon and 94% also spoke Mon. People who identified themselves as Karen and spoke the Karen language made up less than 2% (Table 1).

### Duration of residence and work history in Thailand

Almost all migrants had come to Thailand for the purpose of finding work (Table 2). Migrants from Myanmar had a longer mean duration of residence in Thailand than migrants from Cambodia, with a median of 23 months for M1 from Cambodia and a median of 72 months for M1 from Myanmar. Compared to M1 migrants from Cambodia, M1 migrants from Myanmar were more likely to have family members with them (78% vs. 60%). Cambodian M1 and M2 migrants did not differ with respect to the proportion accompanied by family members. Migrants from Myanmar were also more likely to own a home in Thailand; 35% of M1 from Myanmar owned a home compared to 5% of Cambodian M1.

**Table 2:** Weighted analysis of living status of migrants in Thailand

Variables	Cambodian		Myanmar	
	
	M1* n = 350% (95% CI)	M2 ** n = 475% (95% CI)	M1 * n = 871% (95% CI)	M2 ** n = 19% (95% CI)
**Purpose of being in Thailand**				
Came for work	95.4% (93.0-97.8)	98.5% (97.8-99.9)	93.1% (90.5-95.3)	100%
Came with family member	2.5% (1.1-3.5)	1.6% (1.1-2.8)	6.2% (4.0-8.6)	---
Came with friends	1.6% (1.1-2.8)	---	0.8% (0.2-1.5)	----
**Duration of stay in Thailand (months)**				
Mean	61.5	2.6	86.8	4.5
Median(IQR)	23(12-60)	2(1-3)	72(33-113)	4 (3-5)
Min-Max	7-724	1-6	7-564	2-6
**Own home in Thailand**	5.3% (3.0-8.7)	0.5% (0.0-1.3)	35.0% (31.0-40.0)	26.0% (3.8-51.5)
**Location of family members**				
With them in Thailand	59.8% (50.2-67.3)	57.0% (52.0-62.3)	77.7% (74.2-81.0)	63.6% (32.6-88.5)
Elsewhere in Thailand	0.4% (0.0-0.8)	----	2.9% (2.0-4.1)	---
In Cambodia	39.9% (32.3-47.1)	38.3% (33.0-42.5)	---	---
In Myanmar	---	---	2.0% (1.2-2.9)	---
Other country	---	---	12.9% (10.0-15.8)	29.1% (5.5-55.1)
**Size of family/relatives in Thailand**				
Mean	2.3	1.5	2.4	1.4
Median(IQR)	2(0-4)	1(0-2)	2(1-3)	1(0-3)
**Reasons for not working in home country**				
Grew up in Thailand	---	---	11.3% (8.4-14.2)	---
No jobs in home country	76.5% (71.9-83.1)	76.9% (73.5-81.9)	84.9% (81.6-87.7)	94.4% (82.5-100)
Jobs were irregular	40.7% (34.5-48.4)	51.4% (45.1-56.2)	50.3% (45.9-54.0)	63.5% (38.8-92.9)
Work seasonal jobs in both	3.5% (1.4-5.6)	1.4% (0.6-2.0)	---	---
To be with family	3.5% (1.7-5.1)	---	5.6% (4.2-7.2)	3.3% (0.0-10.9)
**Job/work at initial entry**				
Agriculture	54.4% (43.7-64.6)	91.9% (89.0-94.5)	2.1% (1.2-3.1)	---
Rubber tapping	4.2% (1.8-6.5)	0.9% (0.2-1.8)	88.6% (86.1-91.0)	83.0% (61.4-100)
Domestic	7.5% (4.3-10.6)	0.4% (0.0-0.9)	1.5% (0.6-2.7)	3.4% (0.0-11.4)
Construction	6.6% (3.8-9.0)	0.6% (0.0-1.0)	5.6% (3.8-7.7)	---
Fishing	12.1% (8.4-15.5)	0.9% (0.4-1.4)	2.3% (1.3-3.4)	---
Mining	---	---	0.9% (0.5-1.4)	---
Forestry	0.3% (0.1-0.7)	---	---	---
Restaurant/shop	5.1% (2.5-8.8)	2.5% (0.9-4.0)	0.8% (0.3-1.4)	---
Factory	5.7% (3.1-7.7)	---	3.4% (2.3-4.6)	8.9% (0.0-21.3)
**Benefits received from employer**				
Health insurance	0.2% (0.0-0.6)	1.1% (0.0-2.0)	8.2% (6.4-10.6)	9.2% (0.0-25.4)
Salary	12.1% (7.9-17.5)	10.8% (7.1-14.2)	---	---
Food	6.8% (3.0-10.7)	4.4% (2.3-7.3)	---	---
Water	7.1% (3.5-11.6)	8.1% (5.0-10.8)	---	---
Housing	8.0% (4.3-12.7)	9.1% (5.5-11.9)	0.4% (0.1-0.8)	---
**Duration of being in this job/work(Years)**				
Mean	3.2	***	5.5	0.4
Median(IQR)	2(1-3)	0.2(0.1-0.3)	5(2-7)	0.4(0.2-0.5)
**Plan to continue in this job**				
Until the job ends	20.2% (14.3-24.8)	31.4% (27.1-35.5)	13.7% (11.2-16.3)	8.6% (0.0-20.1)
Don't know/no plans to leave	68.5% (62.1-75.3)	45.9% (42.1-51.2)	83.9% (81.7-87.1)	73.8% (59.6-100)

IQR: Interquartile range

* Has lived in Thailand for 6 or more months; ** Has lived in Thailand for less than 6 months

The primary reason given by all migrant groups for seeking work outside the home country was lack of jobs there (Table 2). Of M1 migrants from Myanmar, 11% had grown up in Thailand. Types of employment varied by country of origin and duration of residence in Thailand. The majority of migrants from Myanmar worked in rubber tapping (89% of M1 and 83% of M2), though small proportions of M1 worked in other fields (construction - 5.6%, factories - 3.4%, and fishing - 2.3%). Of Cambodian M2, 92% worked in agriculture, while Cambodian M1 worked in a greater variety of industries: agriculture - 54%, fishing - 12%, domestic labour - 7.5%, construction - 6.6%, factories - 5.7%, and restaurants/shops - 5.1%. Duration in the current job was longer for M1 migrants from Myanmar than from Cambodia, with a median duration of 5 years vs. 2 years. Of M1 migrants, 84% of those from Myanmar and 69% of those from Cambodia had no plans to leave, and an additional 14% of those from Myanmar and 20% of those from Cambodia planned to continue until the job ended. Only 46% of Cambodian M2 had no plans to leave, and 31% planned to stay until the job ended, leaving 23% with definite plans to leave.

### Health care-seeking behaviour and health messages

A remarkably high proportion (98%) of M1 migrants from Myanmar reported having health insurance, compared to 15% of Cambodian M1 and 3.4% of Cambodian M2. For treatment of the most recent illness episode, 63% of M1 from Myanmar went to a government clinic, compared to 42% and 45% of Cambodian M1 and M2 respectively. Cambodians were more likely to go to a pharmacy (18% and 10% for M1 and M2 respectively), private clinic (10% and 4% for M1 and M2 respectively), or self treat (7% and 11% for M1 and M2 respectively), while only 3% of those from Myanmar used all these options combined. Thirty-one percent of Myanmar M1, 15% of Cambodian M1, and 27% of Cambodian M2 sought no treatment. While no migrants from Myanmar reported returning to Myanmar for treatment, the majority of migrants from Cambodia who sought treatment returned to Cambodia for treatment. Among M2 migrants, 72% who went to a government clinic, 94% who went to a private clinic, and 87% who went to a pharmacy did so in Cambodia. Cambodian M1 migrants were less likely to return to Cambodia for treatment of the last illness, but a substantial minority did so (19% of those who went to a government clinic, 47% who went to a private clinic, and 48% who went to a pharmacy). The primary reason in all groups for choosing a site for treatment was proximity, followed by price. For those that did not seek treatment, the primary reason among Cambodians was preferring self-treatment, while among those from Myanmar, it was felt to be a minor illness that did not necessitate treatment (Table 3).

**Table 3:** Weighted analysis of behaviors of migrants in seeking health care and health messages

Variables	Cambodian		Myanmar	
	
	M1* n = 350 (95% CI)	M2** n = 475 (95% CI)	M1* n = 871 (95% CI)	M2** n = 19 (95% CI)
**Has Thai health insurance**	14.7% (10.9-19.3)	3.4% (1.4-5.4)	98.7% (97.3-99.7)	100%
**Treatment place of last sickness episode**				
No treatment sought	15.2% (10.3-19.6)	27.3% (23.9-32.3)	30.9% (26.8-34.2)	52.1% (5.0-71.6)
To government clinic	42.3% (37.1-51.1)	45.1% (40.5-49.6)	62.6% (59.7-67.3)	47.9% (28.7-95.2)
To private clinic	10.2% (6.3-15.1)	4.0% (2.2-5.6)	1.6% (0.9-2.5)	---
Pharmacy	18.0% (12.3-22.3)	10.1% (7.0-12.7)	1.1% (0.1-2.1)	---
HCW at work	---	---	3.5% (2.1-5.2)	---
Self-treat	7.0% (3.0-10.5)	11.3% (8.6-14.3)	0.2% (0.0-0.4)	---
**Of those who sought treatment,% returned to home country**				
Government clinic	19.1% (10.7-27.6)	72.4% (65.3-79.5)	---	---
Private clinic	46.5% (19.6-73.5)	94.3% (85.7-100)	---	---
Pharmacy	47.7% (40.0-61.4)	87.0% (77.0-97.0)	---	---
**Reasons for choosing the treatment site**				
Close	57.2% (49.7-64.2)	42.9% (38.5-47.6)	41.7% (37.6-45.4)	25.1% (4.5-52.7)
Cheap	20.2% (14.9-24.6)	10.3% (7.9-13.1)	22.2% (19.2-25.1)	19.1% (1.5-42.1)
Familiarity	10.8% (6.9-14.7)	3.2% (1.4-5.7)	2.3% (1.4-3.5)	5.1% (0.0-16.2)
Insurance works there	1.6% (0.3-3.2)	1.7% (0.6-2.9)	---	---
Translator present	1.9% (0.6-3.2)	---	---	---
Other	11.3% (4.6-19.2)	6.6% (4.0-8.9)	3.6% (2.4-5.2)	---
**Why did you not seek treatment?**				
Too far	4.8% (2.7-7.1)	1.6% (0.4-3.0)	0.3% (0.0-0.6)	3.9% (0.0-10.1)
Expensive	10.9% (5.1-19.3)	3.5% (1.9-5.2)	0.6% (0.1-1.2)	---
Worried about deportation	5.9% (3.7-7.6)	0.9% (0.3-1.5)	0.3% (0.0-0.7)	---
Had to work	2.3% (0.7-4.8)	2.5% (1.0-4.2)	1.8% (0.9-2.8)	---
Didn't know where	3.0% (1.2-5.3)	9.2% (6.3-11.5)	---	---
Preferred self-treatment	25.1% (17.7-28.1)	27.9% (24.0-32.3)	6.1% (4.5-7.7)	3.9% (0.0-10.4)
It was a minor illness	19.4% (14.1-24.4)	13.3% (9.8-16.6)	31.7% (28.5-35.2)	35.4% (15.7-67.8)
Too far	4.8% (2.7-7.1)	1.6% (0.4-3.0)	0.3% (0.0-0.6)	3.9% (0.0-10.1)
**Received health messages in past 3 month**	42.8% (36.0-51.3)	13.3% (9.6-16.5)	77.4% (73.7-80.5)	24.4% (6.6-49.6)
**Primary channels**				
Family/friends	3.9% (1.9-6.3)	0.5% (0.0-1.2)	6.8% (5.4-8.3)	---
Health care worker	16.8% (11.3-25.1)	8.0% (5.5-12.8)	50.1% (46.0-54.1)	8.8% (0.0-21.5)
Billboard	2.3% (0.8-4.2)	0.3% (0.0-0.7)	21.2% (17.9-24.9)	20.0% (0.0-43.9)
Radio	8.4% (5.3-11.3)	3.2% (1.7-4.7)	1.1% (0.4-1.9)	---
TV	28.0% (21.3-32.8)	6.4% (4.0-8.6)	30.7% (26.9-36.7)	10.6% (0.0-26.6)
Brochures	---	---	10.3% (8.5-14.6)	5.0% (0.0-15.3)
Other	5.4% (2.5-8.3)	4.05 (1.8-6.2)	0.8% (0.2-1.5)	
**Primary locations**				
Home	30.5% (23.2-35.5)	8.1% (5.5-11.0)	70.2% (66.4-74.2)	17.7% (5.5-37.9)
Market	3.1% (1.7-4.3)	0.1% (0.0-0.3)	---	---
Work place	5.3% (2.8-7.8)	2.4% (0.8-4.0)	2.1% (1.1-3.3)	
Clinic	7.5% (2.7-15.3)	1.2% (0.5-2.0)	14.1% (10.9-17.3)	6.2% (0.0-20.5)
Border crossing	1.0% (0.0-2.5)	0.3% (0.0-1.1)	---	---

* Has lived in Thailand for 6 or more months; ** Has lived in Thailand for less than 6 months

Migrants from Myanmar were most likely to have received health messages in the last 3 months (77%), followed by Cambodian M1 (43%) and Cambodian M2 (13%). Migrants from Myanmar received messages primarily from health care workers (50%), television (31%), and billboards (21%), and brochures (10%), while Cambodians received them from television (28% of M1 and 6% of M2), health care workers (17% of M1 and 8% of M2), and radio (8% of M1 and 3% of M2). Health messages were received at their residences by 70% of Myanmar M1, 31% of Cambodian M1, and 8% of Cambodian M2.

### Knowledge, perception, and practice regarding malaria

The majority of migrants had heard of malaria, with Cambodian and Myanmar M1 (75% and 80%, respectively) more likely to have heard of malaria than Cambodian M2 (55%). Approximately one-third of each group reported either themselves or a family member had experienced malaria; there was no difference between migrants from Cambodia or Myanmar, or among M1 or M2. Of Cambodians, 3.2% of M1 and 7.4% of M2 reported having been treated for malaria in the past three months, compared to 0.6% of Myanmar M1. Cambodians were not significantly different from each other in this respect, but were different from Myanmar migrants. Of Cambodians, 98% of both M1 and M2 lived in households that owned one or more bed nets, and 95% of M1 and 97% of M2 slept under one, compared to 92% of migrants from Myanmar who lived in households that owned one or more bed nets, and 90% that slept under one (Table 4).

**Table 4:** Weighted analysis of knowledge, perception, and practices towards malaria of migrants

Variables	Cambodian		Myanmar	
	
	M1 * (n = 350) (%)	M2** (n = 475) (%)	M1 * (n = 871) (%)	M2 ** (n = 19) (%)
**Heard of malaria**	75.0% (69.4-81.7)	54.7% (49.8-59.3)	80.0% (76.6-83.3)	48.4% (21.9-75.7)
**Self or family member has had malaria**	35.3% (29.9-42.6)	30.4% (25.3-33.6)	29.9% (26.3-34.8)	11.4% (0.0-37.5)
**Treated for malaria in the past 3 months**	3.2% (1.1-5.6)	7.4% (4.7-9.9)	0.6% (0.2-1.1)	5.3% (0.0-23.1)
**Owns one or more bed nets**	97.8% (95.8-99.7)	98.4% (96.7-99)	92.0% (89.8-93.8)	84.4% (62.2-100)
**Slept under a bed net the previous night**	94.6% (91.1-98.2)	97.2% (95.0-99.5)	90.4% (87.9-92.8)	86.6% (68.4-100)
**How malaria is transmitted**				
Mosquitoes	76.0% (70.4-82.2)	63.4% (58.6-68.2)	83.9% (81.0-87.0)	52.2% (26.3-82.6)
Water	4.8% (2.3-7.3)	2.0% (0.8-3.3)	---	---
Work in forests	8.5% (5.6-12.0)	3.9% (2.4-5.3)	0.3% (0.0-0.8)	---
Don't know	19.6% (13.9-25.0)	33.2% (28.6-37.8)	15.2% (12.0-18.2)	37.0% (9.2-64.7)
**Malaria symptoms**				
Fever	64.4% (57.8-71.3)	40.9% (35.8-46.0)	56.7% (52.9-60.8)	28.4% (8.0-55.3)
Sweats	11.8% (9.1-16.7)	17.6% (14.2-21.1)	22.3% (18.0-24.3)	1.6% (0.0-3.9)
Body aches	12.1% (9.0-16.1)	7.1% (5.3-9.7)	29.7% (26.5-33.3)	20.1% (3.6-46.5)
Headache	72.2% (66.6-78.2)	50.3% (45.5-55.2)	75.9% (72.5-79.3)	37.9% (15.2-65.6)
Anorexia	3.6% (1.9-5.5)	3.8% (2.2-5.8)	11.4% (7.5-12.7)	1.7% (0.0-4.8)
Diarrhoea	2.5% (1.0-4.9)	0.5% (0.1-1.0)	0.1% (0.0-0.3)	---
Convulsions	1.3% (0.5-2.3)	---	0.2% (0.0-0.5)	---
Dizziness	10.8% (7.5-14.1)	4.05 (2.3-6.0)	16.6% (13.8-19.7)	11.9% (0.0-32.1)
Chills	68.2% (62.7-74.7)	57.9% (53.1-62.6)	57.7% (54.1-61.4)	25.6% (5.9-50.9)
Other	10.7% (6.7-15.2)	12.8% (9.9-16.6)	13.7% (10.6-17.0)	35.4% (0.0-62.0)
Don't know	11.1% (6.1-15.0)	31.0% (26.2-35.9)	6.6% (5.1-8.4)	11.8% (2.5-28.3)
**Malaria prevention**				
Mosquito net	85.6% (80.7-90.6)	65.5% (60.5-70.7)	87.2% (84.6-89.9)	56.2% (30.0-89.1)
Prophylactic medicine	2.6% (0.8-5.5)	4.3% (2.6-5.6)	0.2% (0.1-0.4)	---
Spray repellant	29.9% (24.8-37.6)	14.6% (12.1-18.4)	76.2% (72.6-79.4)	46.7% (24.0-83.4)
Mosquito coils	23.4% (17.7-29.4)	5.8% (3.8-8.4)	41.1% (37.6-45.3)	1.5% (0.0-4.1)
Keep house clean	3.2% (1.7-5.0)	7.5% (5.1-11.0)	---	---
Cover water tanks	3.1% (1.6-4.6%)	1.5% (0.7-2.6)	0.1% (0.0-0.3)	---
Close windows/door	1.9% (0.4-3.0)	3.8% (1.7-5.7)	---	---
Other	6.1% (3.0-8.8)	3.4% (1.9-5.2)	7.6% (5.6-10.0)	19.1% (0.0-36.5)
Don't know	13.7% (8.7-18.6)	32.0% (27.6-36.5)	9.4% (7.0-11.9)	30.0% (0.7-58.6)
**Malaria treatment**				
Medicine from health worker	63.3% (55.5-70.7)	40.9% (36.2-45.3)	53.3% (46.6-60.3)	22.1% (5.6-53.2)
Herbal medicine	4.5% (2.4-7.1)	3.5% (1.7-5.0)	0.1% (0.0-0.2)	---
Prayer/meditation	---	---	---	---
Other	3.3% (1.2-5.7)	5.7% (3.2-8.9)	34.5% (28.6-43.0)	32.7% (5.2-68.3)
Don't know	25.8% (18.0-33.5)	50.1% (45.5-55.2)	5.8% (3.9-7.9)	33.6% (5.0-60.9)

* Has lived in Thailand for 6 or more months; ** Has lived in Thailand for less than 6 months

The majority knew that malaria is transmitted by mosquito, with knowledge of M1 migrants from Cambodia and Myanmar similar (76% and 84%, respectively), and greater than Cambodian M2 (63%). The primary symptoms of malaria mentioned by both nationalities were headache, chills, and fever; 31% of Cambodian M2 did not know any symptoms, compared to 11% of Cambodian M1 and 7% of Myanmar M1. While mosquito nets were the most frequently mentioned method of prevention, spray repellant and mosquito coils were popular among both; approximately one-third of M2 from Cambodia and Myanmar did not know, compared to 14% of Cambodian M1 and 9% of Myanmar M1. In terms of best malaria treatment, among Cambodians, 63% of M1 chose medicine obtained from a health care worker and 26% did not know, while among M2, only 41% chose medicine from a health care worker and 50% did not know. Among M1 migrants from Myanmar, 53% chose medicine from a health care worker, while 35% chose other methods not described, and only 6% didn't know. None chose prayer/meditation, and less than 5% of Cambodians and 0.1% from Myanmar chose herbal medicine (Table 4).

## Discussion

Among migrant workers in Trat, Chantaburi, and Sa Kaeo provinces on the Thailand-Cambodia border, substantial differences exist between migrants who have been in Thailand for less than six months and longer-term migrants, and between migrants from Cambodia and those from Myanmar. These differences must be taken into consideration when designing malaria control strategies.

Cambodian migrants were more likely to be short-term workers, male, under 25 years of age, with little formal education, very little knowledge of spoken or written Thai, and working in the agricultural sector. Longer-term workers from Cambodia had more access to education and a majority spoke Thai, though few read it, and there was a broader range of occupations. Migrant agricultural workers typically work and sleep on one farm, dependent on the farm owner for access to services, and thus may have limited access to health services and messaging. Many would likely not benefit from radio or TV messages in Thai heard at their residences or workplaces. Migrants from Myanmar had a longer duration of residence in Thailand, and while their level of formal education was similar to Cambodian M2, the majority spoke Thai, though few read it. While most worked in rubber tapping, about one-third owned their own home and were more integrated within the community, with greater access to health services and messaging.

Consequently, knowledge of malaria differed among migrant groups. While the majority of long-term migrants are knowledgeable about malaria transmission, prevention, and treatment, a consistent one-third of short-term migrants from Cambodia reported little to no knowledge of these factors, yet had the highest proportion of persons reported treated for malaria in the previous 3 months. While most cited bed nets as a preventive measure, and the overwhelming majority of migrant workers from Cambodia and Myanmar own and sleep under bed nets, the proportion that knew to get anti-malarial medicines from a health care worker was comparatively low. Long-term migrants were more likely to have received health messages than short-term migrants, and those from Myanmar more likely than Cambodians. Migrants from Myanmar were much more likely to receive messages from a health care worker than Cambodian migrants, perhaps due to the higher use of public health facilities.

These results suggest the need for more targeted and effective health messaging in migrant communities, especially in among short-term migrants from Cambodia. Given the low level of Thai literacy, oral media, such as health care workers, television, and radio were the most effective mediums used. Presenting oral material in native languages, such as Khmer, may be the most effective strategy to reach short-term migrants. While brochures reached very few, levels of Khmer literacy among Cambodians and Burmese literacy among those from Myanmar indicate that using printed material in the native language may be effective. Well-developed and evidence-based Information, Education and Communication (IEC) and Behaviour Change Communication (BCC) materials are needed to increase knowledge in the community of symptoms, prevention and control measures, sources of treatment and care and the risks associated with delays in treatment [9]. Strategies and materials need to be based on the needs, characteristics, and culture of the migrant workers in the areas [17-23].

Clearly, access to health insurance influences health care-seeking patterns; most migrant workers from Myanmar have health insurance in Thailand, and not surprisingly are more likely to use government health facilities than Cambodians, a majority of whom do not have health insurance and return to Cambodia to seek care. Short-term migrants are even more likely than long-term migrants to return to Cambodia for health care, and have a high rate of self-treating or not seeking treatment.

The most important determinants of treatment-seeking behaviour for both nationalities were proximity and cost. Access is a concept involving awareness of people's need for medical care service, availability of services and acceptability of the service and affordability to the service [24]. Regulations and policy for malaria control and prevention were different and continuously evolving between Thailand and Cambodia; for example the issues of case management and free access to health care services [25]. Previous studies in the region have reported factors associated with health care services access and utilization. In a study on health-seeking behaviours among Myanmar migrant workers in southern Thailand [26], buying drugs from a drug store was the most common health-seeking behaviour when the health problem was perceived to be minor, but care was sought at health centers for health problems perceived to be major. The choice among the available options was determined by the availability of health facilities, cost fees, satisfaction with services, accessibility, knowing where and how to obtain health services, and belief in traditional medicine.

Community-based interventions and services through a network of village health workers (VHWs) and community volunteers to strengthen malaria prevention and control measures may be particularly useful for the Cambodian migrants that have little access to health services [27,28]. In Thailand, IOM has piloted several field projects to develop the Migrant Health Programme Model [29]. The model further promotes migrant community volunteers who are in a position to culturally interact with migrants, promote good health practices and collaborate with Thai health workers to increase access to and use of basic health services, including malaria services, by migrants. Migrant health teams, which are part of the model, are also set up at district and provincial levels to ensure that challenges are discussed, addressed and monitored at policy decision levels.

It will be essential for the BVBD and the Cambodian National Malaria Programme (CNM) to work together on effective cross-border strategies taking into account the migrant's level of education, language, usual point of access of health messaging, and mobility. It is also essential that these strategies include employers as for many migrants, work is their only point of access to health messaging and services. This will necessitate the development of a strategy with the Ministry of Labour to approach employers who are using undocumented workers in a non-threatening way. While core universal approaches to prevention and control measures for all migrants along the border should be utilized, different approaches and strategies should also be planned for each respective group in order to reach effectiveness in major goals for disease containment or elimination. Changes in population distribution and migration trends should be taken into consideration. The IEC/BCC development and malaria control efforts should identify how to refine, simplify and scale up replicable interventions that will add value and impact to a regional concept rather than just country-specific plans, and should not ignore vulnerable and hard-to-reach ethnic minority populations.

In addition to the activities outlined above, medical insurance and assistance programme options are under consideration in Thailand to provide affordable health care services to all migrants [26,30,31]. In addition, advocacy combining social networking and mobilization, interpersonal communication and negotiation, as well as the use of media for generating public pressure might be effective tools for health care professionals to make sustainable social change [32].

## Conclusion

The study made possible the characterization of demographic information, migratory patterns, knowledge, perceptions, health behaviours and practices of short and long-term migrants from Myanmar and Cambodian living on the Thailand-Cambodian border. These findings are concerning, particularly that the most mobile migrants have low access to health messages, are not accessing proper malaria diagnostics and treatment, and are carrying potentially resistant parasites when they return to their homes to seek treatment. This information has great potential to help determine more effective communication and health outreach strategies for the containment project. Cross-border communication and collaboration will be necessary to effectively implement these strategies.

## Competing interests

The authors declare that they have no competing interests.

## Authors' contributions

PW, CD, WS, AK, and JE were involved in the conception and design of the study and design of the application tools for data collection. PW was in charge of managing the study and monitoring field research activities. CD arranged for technical assistance and consultation of the study conceptual framework. WS was responsible for managing and supervising overall malaria control programme activities. AK monitored field activities under RDS method and extracted data for analysis. PW, AK, JT and JK performed statistical analyses and drafted the manuscript. All authors read and approved the final manuscript.
